# Integrating addiction treatment into primary care using mobile health technology: protocol for an implementation research study

**DOI:** 10.1186/1748-5908-9-65

**Published:** 2014-05-29

**Authors:** Andrew R Quanbeck, David H Gustafson, Lisa A Marsch, Fiona McTavish, Randall T Brown, Marie-Louise Mares, Roberta Johnson, Joseph E Glass, Amy K Atwood, Helene McDowell

**Affiliations:** 1 Center for Health Enhancement Systems Studies, Industrial and Systems Engineering Department, University of Wisconsin – Madison, Madison, WI 53706, USA; 2 Center for Technology and Behavioral Health, Dartmouth Psychiatric Research Center, Dartmouth College, Lebanon, NH 03766, USA; 3 Center for Addictive Disorders, School of Medicine and Public Health, University of Wisconsin – Madison, Madison, WI 53713, USA; 4 Communication Arts Department, University of Wisconsin – Madison, Madison, WI 53706, USA; 5 School of Social Work, University of Wisconsin – Madison, Madison, WI 53706, USA

**Keywords:** Primary care integration, Systems change, E-Health, RE-AIM framework

## Abstract

**Background:**

Healthcare reform in the United States is encouraging Federally Qualified Health Centers and other primary-care practices to integrate treatment for addiction and other behavioral health conditions into their practices. The potential of mobile health technologies to manage addiction and comorbidities such as HIV in these settings is substantial but largely untested. This paper describes a protocol to evaluate the implementation of an E-Health integrated communication technology delivered via mobile phones, called Seva, into primary-care settings. Seva is an evidence-based system of addiction treatment and recovery support for patients and real-time caseload monitoring for clinicians.

**Methods/Design:**

Our implementation strategy uses three models of organizational change: the Program Planning Model to promote acceptance and sustainability, the NIATx quality improvement model to create a welcoming environment for change, and Rogers’s diffusion of innovations research, which facilitates adaptations of innovations to maximize their adoption potential. We will implement Seva and conduct an intensive, mixed-methods assessment at three diverse Federally Qualified Healthcare Centers in the United States. Our non-concurrent multiple-baseline design includes three periods — pretest (ending in four months of implementation preparation), active Seva implementation, and maintenance — with implementation staggered at six-month intervals across sites. The first site will serve as a pilot clinic. We will track the timing of intervention elements and assess study outcomes within each dimension of the Reach, Effectiveness, Adoption, Implementation, and Maintenance framework, including effects on clinicians, patients, and practices. Our mixed-methods approach will include quantitative (*e.g*., interrupted time-series analysis of treatment attendance, with clinics as the unit of analysis) and qualitative (*e.g*., staff interviews regarding adaptations to implementation protocol) methods, and assessment of implementation costs.

**Discussion:**

If implementation is successful, the field will have a proven technology that helps Federally Qualified Health Centers and affiliated organizations provide addiction treatment and recovery support, as well as a proven strategy for implementing the technology. Seva also has the potential to improve core elements of addiction treatment, such as referral and treatment processes. A mobile technology for addiction treatment and accompanying implementation model could provide a cost-effective means to improve the lives of patients with drug and alcohol problems.

**Trial registration:**

ClinicalTrials.gov (NCT01963234).

## Background

### Context

This paper describes the protocol for a study on implementing mobile-health technology in primary care. The study is funded by the United States’ National Institute on Drug Abuse as part of a request for proposals aimed at integrating addiction treatment into primary care*.* Although numerous smartphone applications exist that address many health problems, primary care clinicians do not commonly incorporate mobile technology into patient care [1]. One reason is that very few smartphone applications have been proven in rigorous trials of effectiveness. Even if an application does have a reliable evidence base, it must still demonstrate its value to various stakeholders if it is to be widely used. Patients must see value in the application or they are likely to stop using it. Clinicians must find it useful to incorporate it in day-to-day clinical management. Finally, clinic administrators must find that the application works both clinically and financially to support its use by patients and staff.

Our study focuses on Federally Qualified Health Centers (FQHCs) in the United States, community-based organizations that provide primary care and preventive health services to patients regardless of their ability to pay. FQHCs (and U.S. primary-care practices in general) are increasingly being encouraged by healthcare reform to integrate treatment for addiction and behavioral health into their practices. HIV testing and prevention efforts are particularly important for the health of men and women who use illicit drugs because they are at higher risk than the general population for acquiring HIV [2,3]. But transforming primary care to address addiction and HIV will likely add to the work of clinicians, many of whom already feel overburdened. Previous attempts to integrate addiction treatment into primary care have largely relied on labor-intensive solutions (*e.g*., adding more staff or having staff do tasks differently), with mixed results [4].

Our premise is that mobile health technology holds great promise for FQHCs to reach this complex patient population. More than two-thirds of individuals with substance use disorders (SUDs) have had contact with a primary-care provider in the previous six months [5], for reasons that may or may not relate to their SUD [6]. But primary-care providers frequently do not screen for or treat SUDs, even though recovery support can be effectively administered in primary care [6,7]. Administering recovery support requires that clinicians receive support and training in addiction, which are uncommon partly because of the traditional separation of addiction treatment from primary care [8-10]. Technology may help address these problems. People with addictions tend to view technology favorably [11]. They acknowledge more drug use and psychiatric symptoms online than in face-to-face interviews [12]. A recent review found positive outcomes in 29 of 32 randomized trials using personal computers and cell phones for managing chronic diseases [13]. While some information technologies have proven efficacious, penetration into practice has been slow [14]. The potential of mobile health technologies to manage chronic diseases in FQHCs is substantial, but remains largely untested.

### The mobile-health technology

The integrated system we have developed is an E-Health integrated communication technology for patients with addiction called Seva (say’-va; a Sanskrit word for selfless caring). Seva consists of treatment and recovery support elements. The treatment component is based on the Therapeutic Education System (TES), a web- and mobile-based curriculum for addiction treatment with 65 interactive, multimedia, skill-building modules, including basic cognitive behavioral recovery support skills (*e.g*., refusing drugs, managing thoughts about drug use), life re-structuring skills (*e.g*., increasing recreational activities), and skills for preventing HIV, hepatitis, and sexually transmitted infections. Each module ends with a quiz structured to ensure mastery of key content and skills. TES uses interactive videos to create experiential learning environments that help patients learn modeled behaviors (*e.g*., progressive muscle relaxation). Randomized trials have found that TES is as efficacious as behavioral therapy delivered by therapists and is superior to standard substance abuse treatment [15]; that it increases HIV prevention knowledge and self-reported risk for HIV [16]; and when it partially replaces clinician-delivered care in methadone treatment, it also improves opioid abstinence rates [17].

Seva’s recovery support elements are based on A-CHESS, an evidence-based smartphone program designed to help people in recovery from drug and alcohol addiction prevent relapse. A-CHESS helps patients meet the challenges they often face, such as loneliness and isolation, transportation, managing the treatment regimen, and getting informal support. A-CHESS also addresses issues such as cravings and insufficient coping skills in high-risk situations. A-CHESS is based on research on the principles of effective continuing care for substance use disorders (long duration, assertive outreach, monitoring, prompts, action planning, peer and family support, and case management). The research team that developed A-CHESS has studied and designed similar systems that have been effective for patients suffering from lung cancer, breast cancer, asthma, and HIV [2,3,18-36]. In a randomized trial of patients discharged from residential addiction treatment, patients with A-CHESS had 57% fewer risky drinking days than patients in the control group [37].

Although Seva was built from two existing programs, TES and A-CHESS, the Seva user experiences one seamless system with many links between elements. For example, upon completing a skill-building module, the patient may be shown a link to a discussion group where peers are discussing the topic of the module, and provided links to further information on the topic. Similarly, patients whose responses to check-in items indicate a potential problem may see a link to skill-building related to that problem, or be shown personal stories from other patients who have dealt with the same problem. Patient-entered data are also uploaded and presented via a web-based dashboard that FQHC clinicians can use to monitor the status of their Seva users. The dashboard includes items that patients self-report using their mobile devices, such as sleep troubles and relationship problems that may signal an imminent relapse; whether the patient has attended recovery meetings or completed other elements of the treatment plan; and the patient’s relapse risk and protection factors. The dashboard can be customized for each clinic to display indicators that clinicians deem important for clinical decision-making.

### Study focus: implementation

Because Seva has an established evidence base, this research focuses mainly on implementation and organizational impact; some patient-level outcomes are included, but they will be examined in the aggregate. Our goal is to implement a system that has already proven effective in specialty addiction treatment in primary-care settings. The implementation model we are testing (detailed below) is distinguished by several characteristics: an effort to actively prepare the environment for implementation; the use of an organizational coach; the use of rapid-cycle tests; and an early, specific effort to address sustainability. The implementation model is rooted in the field of systems engineering, a multi-disciplinary field that integrates concepts from psychology, economics, social science, and statistics. The implementation model reflects the multi-disciplinary ethic of systems engineering.

We will study organizational change resulting from implementing Seva in 3 FQHCs over a three-year period. We will study elements of our implementation model by using mixed-methods analysis. Our study seeks answers to three broad research questions: how can Seva be implemented in primary care settings efficiently and effectively; to what extent do patients and staff accept and use Seva; and how does Seva affect clinical care for patients and staff?

## Methods/Design

### Implementation model

**Figure 1:**
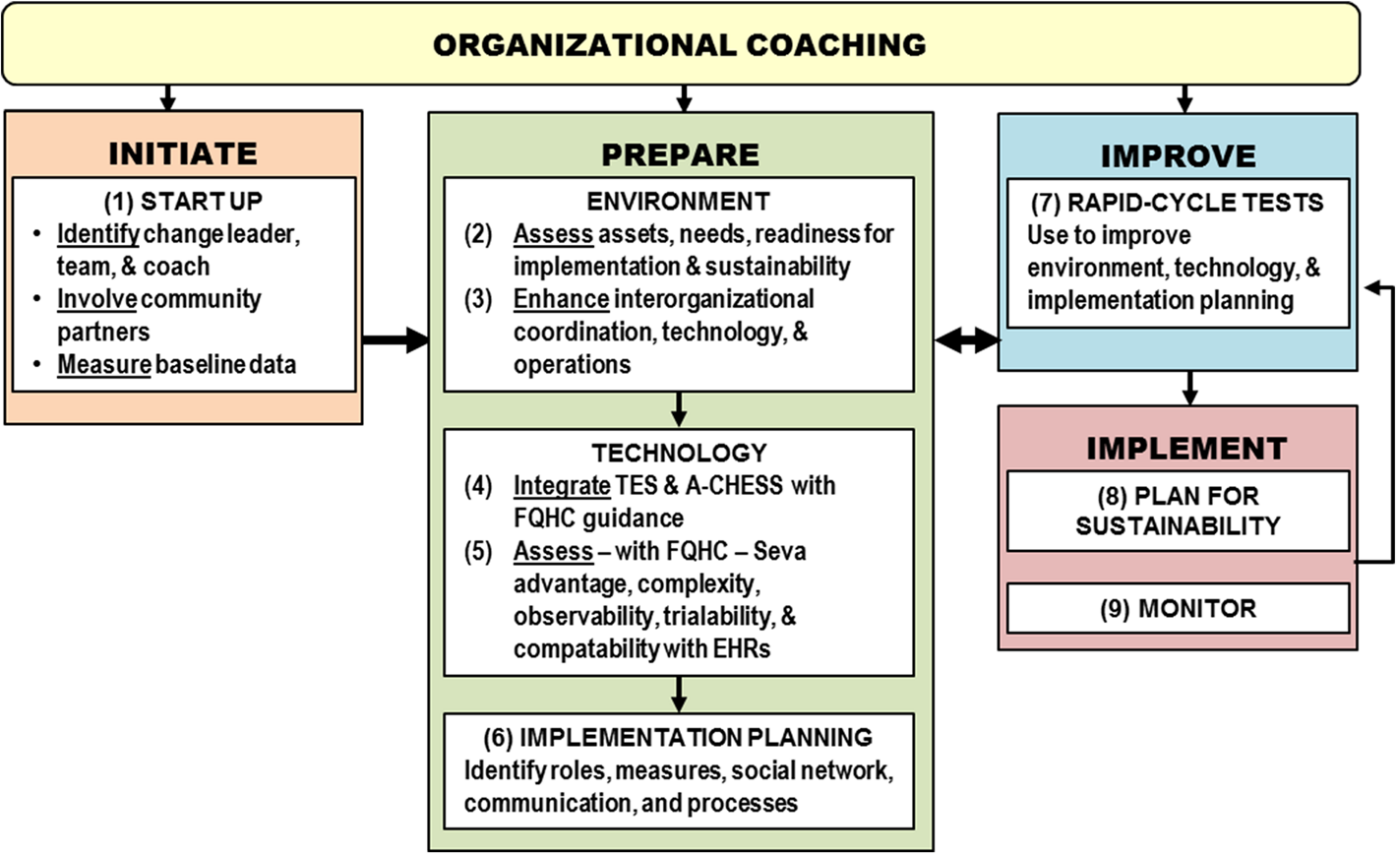
Implementation model.

Our implementation strategy combines three evidence-based organizational change strategies: the Program Planning Model (PPM) [38,39], the NIATx quality improvement model [40,41], and core concepts from Rogers’s diffusion of innovations research [42]. The PPM comes from the literature of organizational behavior; NIATx, from systems engineering; and Rogers, from community-based diffusion. The PPM addresses interpersonal, organizational and environmental variables that promote sustainability. By focusing on socio-political aspects of change in community-based organizations, use of the PPM has led to greater community acceptance than seen in comparison groups [38,39]. The NIATx model, an extensively evaluated and widely adopted organizational change model in addiction treatment, provides an evidence-based approach to implementation by optimizing processes that create a welcoming environment for change [40,41]. Rogers’s diffusion of innovations research identified characteristics of innovations associated with successful implementation and sustainability [42]. Rogers’s research complements the NIATx model by emphasizing ways to adapt an innovation for a specific setting, thus maximizing its adoption potential. Combined, these strategies make up the implementation model shown in Figure 1.

The model has 4 phases: i) ‘Initiate’ involves bringing together and training key people at each site; ii) ‘Prepare’ ensures that the FQHC environment is welcoming to Seva, the implementation plan adapts to the unique characteristics of the FQHC, and the technology is developed to maximize its chance of success; iii) ‘Improve’ involves conducting a series of rapid-cycle tests of ideas that emerge from earlier phases; and iv) ‘Implement’ involves using, monitoring and sustaining the technology. Organizational coaching will play a key role in helping clinics implement Seva successfully. We recently completed a randomized trial in 201 addiction treatment agencies in which coaching proved to be the most cost-effective approach to quality improvement among several strategies tested [43]. An implementation coach will work with each FQHC to advise, encourage and monitor progress.

### Settings for implementation

We worked with the National Association of Community Health Centers to identify a set of FQHCs with established electronic health records. We focused on FQHCs with electronic health records because healthcare reform has been pushing FQHCs in this direction, and we believe it will be important to understand how Seva relates to existing clinic technology. From the pool of FQHCs that met these criteria, we selected an FQHC affiliated with the University of Wisconsin as our first implementation site; as a second site, a relatively small, rural, freestanding FQHC with integrated behavioral health services (including addiction treatment); and as a third site, an urban FQHC that serves a largely minority population. In selecting sites, we strove to maximize diversity in both patient populations and organizational structures to better understand how environment affects implementation. We included only three FQHCs in order to conduct an intensive, mixed-methods assessment at each clinic, which we believe will be more informative for the ultimate purpose of dissemination than randomizing a larger sample of organizations and studying implementation at a surface level.

### Timeline

**Figure 2:**
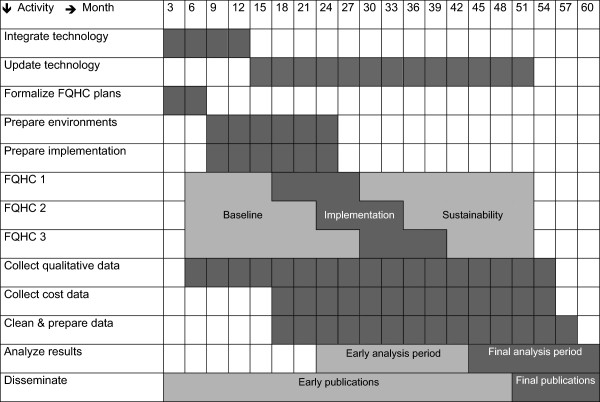
Project timeline.

Data will be collected from each FQHC for 48 months using a combination of data sources, including electronic health records, staff surveys/interviews, and patient surveys/interviews. We are collecting staff survey data every 6 months for 36 months, patient survey data every 6 months for 18 months, and aggregated EHR data for 48 months to cover the full time frame from the beginning of the pretest period at FQHC 1 to the end of the posttest period at the FQHC 3. Figure 2 shows more information about the project timeline. Retrospective data collection will take place during the pretest period, using aggregated patient data from clinics’ electronic health records. During the pretest period, sites will also seek to identify potential patient candidates for recruitment. Full-scale implementation occurs during the subsequent 12 months, during which the sites receive coaching and actively recruit patients to use Seva. The sites will continue to be monitored during a post-test/sustainability period. During this period, sites may continue recruiting patients to use Seva, but without grant funding for phone/internet support or organizational coaching.

### Coaching for organizational change

The organizational coach for the first (pilot) site will be a member of the research team with extensive experience in organizational coaching. The next two sites will be coached by a physician from the pilot site, who will share firsthand experience in implementing Seva with subsequent clinics. Each site will have a designated ‘change leader’, a clinical leader at the site who is the point of contact with the coach and will coordinate implementation activities at the clinic; a ‘site coordinator’, a clinic employee who will enroll patients in the study and manage the day-to-day operation of Seva; and a ‘change team’, a group of four to eight clinical and/or administrative staff members who will help identify and make the organizational changes necessary to implement Seva. The coach will make an initial site visit during the four-month period of active implementation preparation to create a welcoming environment for Seva. During this visit, the coach will conduct a walk-through [44] with change team members (an exercise in which employees experience the clinic as patients do, to reveal issues that need to be addressed); a work flow assessment using flow charting; and a technical assessment of data to be gathered and procedures needed to conduct the study. During this preparation period, the coach will also work with the change leader to ensure that pre-test data are collected; reviews of Seva are completed by clinical teams and their concerns addressed; and barriers to implementation rectified. After the initial site visit, for the remainder of the implementation preparation period and for the 12-month intervention period, the coach will have monthly telephone conferences with clinic staff and email correspondence as necessary to monitor implementation progress and offer advice and feedback to the staff. The coach and change leader will also work together to reduce organizational barriers to implementation.

### Study design

Our study uses a non-concurrent multiple-baseline design in which the start of the intervention period is staggered at the three sites by six months. The multiple-baseline design provides the intervention to all participants, but isolates the independent variable by introducing the intervention at specified points. Using the clinic as the unit of analysis, this single-subject approach will closely track the timing of intervention elements and repeatedly measure outcomes at the organizational level, including aggregated effects on clinicians, patients and practices. After pilot testing Seva at the first FQHC and improving Seva and the overall implementation strategy, we will introduce Seva at the second FQHC and then, six months later, at the third FQHC. Each FQHC acts as its own pretest control. During the 48-month data collection period, core organizational indicators will be measured via a staff survey (administered every 6 months) and the clinics’ electronic health records (aggregated across patients). While the non-concurrent multiple-baseline design may not provide as strong a basis for causal inference as a large RCT, we believe it is the appropriate design for evaluating the organizational impact of a system that has already been shown effective in RCTs of patients [45]. We will also collect a set of standard CHESS/TES measures via a patient survey for comparison to previous studies that tested these technologies in RCTs, and to conduct exploratory analyses. The patient survey also provides supplemental clinical information not routinely collected in the clinics (*e.g*., addiction severity, quality of life, etc.).

### Evaluation framework

Our primary focus is to study how Seva is implemented in three FQHCs. In the process, we hope to generate insights on how Seva can be most helpful to organizations, staff and patients, thereby informing implementation processes in other FQHCs. Our evaluation includes patient, clinician, and organization-level outcomes. To organize this multifaceted evaluation, we use the RE-AIM framework [46]. RE-AIM catalogs the quality, speed and impact of efforts to translate research into practice by including measures in five categories: reach, effectiveness, adoption, implementation and maintenance. The measures we will use are summarized in Table 1, followed by a brief description of each dimension and selected outcomes.

**Table 1:** RE-AIM measures

**Dimension**	**Measure**	**Source**	Research question*	**Frequency**
Reach	Number of Seva patients (eligible, excluded, enrolled)	Patient survey	2	Pre
	Characteristics of participating patients	Patient survey	2	Pre
		EHR	2	Continuous
	Qualitative assessment- reach	Interview- clinic director	1	Pre
Effectiveness	No. patients screened for HIV	EHR	3	Continuous
	Healthcare utilization (hospitalizations, ER visits, and residential addiction treatment)	Patient survey	3	0 m, 6 m, 12 m, 18 m
		EHR	3	Continuous
	Treatment attendance	Patient survey	3	0 m, 6 m, 12 m, 18 m
		EHR	3	Continuous
	HIV risk behaviors	Patient survey	3	0 m, 6 m, 12 m, 18 m
	Substance use	Seva	3	Weekly
		Patient survey	3	0 m, 6 m, 12 m, 18m
	Quality of life	Patient survey	3	0 m, 6 m, 12 m, 18 m
	Qualitative assessment - effectiveness	Interview - clinic director	1	12 m
Adoption (setting)	Characteristics of participating clinics vs. general FQHC population	Publicly available uniform data system reports for FQHCs	2	Pre
	Readiness for implementation	Staff survey	2	Pre, every 6 m
Adoption (staff)	Use of Seva by staff (including characteristics)	Seva log files	2	Continuous
Adoption (patient)	Use of Seva by patients (including characteristics)	Seva log files	2	Continuous
Adoption (staff and patient)	Qualitative assessment- adoption	Staff interviews	1	Pre, every 6 m
		Patient interviews	1	12 m
Implementation	Stages of Implementation Completion	Staff interviews	1	Pre, every 6 m
	Technology acceptance	Staff survey	2	Pre, every 6 m
	Adaptations to protocol during intervention period	Staff interviews	1	Pre, every 6 m
	Cost of intervention	Staff interviews	1	Pre, every 6 m
		Coach logs	1	Continuous
	Qualitative assessment - implementation	Interview with clinic director, coach	1	12 m
Maintenance	Sustainability score	Staff survey	1	Pre, every 6 m
	Six-month follow-up on all effectiveness measures (see above) and use of Seva	Seva, Patient survey, EHR	1, 2, 3	Various
	Qualitative assessment - maintenance	Interview - clinic director	1, 2	18 m

*Source:* Re-aim.org; Measuring the Use of the RE-AIM Model Dimension Items Checklist

*(1) How can Seva be implemented in primary care settings efficiently and effectively? (2) To what extent do patients and staff accept and use Seva? (3) How does Seva affect clinical care for patients and staff?

#### Reach

In assessing reach, we will determine how many patients are eligible to use Seva within each clinic, how many are excluded, how many are invited to participate, and how many enroll in the study. We will also study how quickly patients are enrolled after the beginning of the enrollment period. We will assess how representative Seva enrollees are of the target population (substance using patients within each clinic) by comparing users to the target population on key clinical characteristics (*e.g*., treatment attendance, healthcare utilization). Qualitative assessment will supplement our data collection on reach. Our goal is to understand how clinicians perceive or anticipate which patients may benefit most from the system, because — for budget reasons — not all patients can have Seva, either in this study or in a broader dissemination. (In this study, we can offer Seva to a maximum of 100 patients per clinic.) For example, clinicians may select patients to enroll who they think will be strong models for other patients, or patients who have had many relapses and may benefit from a different treatment approach. As an implementation-focused study, we strive not for random selection but instead encourage clinicians to enroll patients for whom they believe Seva will be most helpful.

#### Effectiveness

Our primary effectiveness outcome is substance use. Other outcomes in the effectiveness domain will help us understand the impact of Seva on patients’ quality of life, HIV risk behaviors, healthcare utilization, and treatment attendance. We will also examine the extent to which the system promotes an overall increase in the proportion of substance-use patients screened for HIV. Healthcare utilization data (hospitalization, emergency room visits, and in-patient psychiatric treatment) will also be used in the cost analysis described below. Qualitative assessment of effectiveness will take place through an interview with the clinic director.

#### Adoption

In assessing adoption at the clinic level, we will report how many FQHCs were approached to participate and how many signed on to implement Seva. We will compare organizational characteristics of participating FQHCs to the more than 1,000 FQHCs in the United States, based on public reports required of these organizations by the federal government. We will gather qualitative data to assess which aspects of organizational structure affect implementation. To assess adoption at the staff level, clinic site coordinators will make a list of all staff members who see potential substance-using patients in primary or behavioral care, and invite these staff to complete surveys six months prior to the implementation period and every six months thereafter. The surveys include questions on job function (*i.e*., physician, behavioral health staff, or RN), which will allow us to assess how representative the respondents are of the clinic staff as a whole, and the Readiness for Implementation Survey [47], an instrument used to predict the adoption success of information technologies in healthcare. Staff members and patients will take part in qualitative interviews to supplement data provided in the surveys to assess organizational barriers and facilitators related to implementation. We will analyze Seva log files to produce metrics about patterns of system adoption and use by patients and staff and assess the characteristics of individuals who use Seva most and least actively.

#### Implementation

To measure implementation progress, we will adapt our implementation model into a specific implementation plan (including timetables) for each clinic. We will employ the Stages of Implementation Completion model [48] as a guide to assess the degree of implementation. Each phase of the implementation completion model is broken down into discrete tasks and events and presented as a checklist. We will assess whether each task/event was implemented, and the length of time each one took to implement. Starting at the beginning of the implementation-planning period (four months prior to implementation), the checklist will be reviewed and updated every six months by the site coordinator in conjunction with the implementation coach. Adaptations to the protocol will be noted. We will also assess implementation costs (see below) and administer the Technology Acceptance Model [49], which helps explain factors that influence the use of a new technology. Qualitative assessment of the consistency of the intervention will occur through interviews with the clinic director and the implementation coach.

#### Maintenance

To assess maintenance, we will administer the British National Health Service’s Sustainability Index [50]. The Sustainability Index assesses factors associated with sustainability potential, such as fit with organizational infrastructure, clinical leadership engagement, and progress monitoring. The Index also offers guidance, developed by an international panel of experts, on overcoming weaknesses. Factors measured by the Sustainability Index, along with those included in the Readiness for Implementation Survey, will guide us in building a conceptual model of barriers and facilitators to implementation. Assessing maintenance at the individual level includes monitoring of effectiveness measures (including substance use and quality of life) at the 18-month mark, 6 months after grant funding for phones and internet access ends. We will examine data on Seva use at the staff and patient levels to assess long-term maintenance of Seva. Qualitative interviews with clinic directors will assess the degree of institutionalization of Seva within organizations, how Seva aligns with clinical business models, and whether and how Seva was adapted after the active implementation period to promote maintenance.

### Analysis

We will use a mixed-method approach, using both quantitative and qualitative methods, to assess our implementation model. Our analysis follows guidelines for mixed-methods research promulgated by the NIH Office of Behavioral and Social Sciences Research [51]. Because this is a study of the organizational impact of Seva, there will be no single-patient analyses; all analyses will be aggregated across patients.

Analysis of quantitative outcomes will proceed on three fronts:

1. Descriptive statistics will be used to assess relevant Reach outcomes.

2. Interrupted time series analysis will compare selected Effectiveness and Maintenance outcomes within a clinic before and after implementation. We will assess whether our implementation model promotes the successful installation and use of Seva using linear regression models, with each analysis having two time-series segments representing means and slopes before and after implementation. A test of the regression coefficient for implementation (a dichotomous variable indicating whether the time point occurred before or after implementation) will allow us to examine differences in the mean change at implementation. Differences in slope from pre- to post-implementation will be tested using the coefficient for the implementation x month interaction, where ‘month’ is a continuous variable indicating the month of measurement. Weighted least squares analysis will be used to account for potential differences in the number of patients or staff reporting outcomes within each clinic across time. Given the limited degrees of freedom, no covariates will be included in the models.

3. Drawing from conventional approaches to single-subject analysis, we will also compare selected RE-AIM outcomes within and across the clinics using data visualization [52] to determine whether the shifts between intervention phases of the study generated shifts in the level, trend, and variability of performance [53]. This will allow us to evaluate the immediacy, consistency and magnitude of the observed effects [52].

The qualitative analysis is designed to:

1. Understand the environments in which Seva is implemented, how Seva was tailored to fit each environment, and the processes used to tailor Seva. Focus groups and observational and interview data will help us understand the environment and the processes used to tailor Seva.

2. Provide insights into the quantitative results. We will interview key stakeholders following each wave of surveys. As within- and across-site findings emerge, we will interview people who can help inform the findings.

3. Understand the barriers to and facilitators of implementing and sustaining Seva and how they relate to outcomes. Key stakeholders will be asked to reflect on reach, outcomes, adoption at the setting level, staff participation, implementation, and long-term effects.

4. Prompt informants to reflect upon how they might advise a similar FQHC to integrate Seva.

We will collect, transcribe and analyze field notes and interviews collected during each implementation phase using AtlasTi. The qualitative analysis will follow grounded theory to build a conceptual model of Seva’s implementation. Two researchers will independently analyze interviews that represent different organization-level stakeholders, and then meet to discuss and reconcile differences in interpretation. Because Readiness for Implementation Survey and Sustainability Index factors are associated with adoption and sustainability, we will include these factors as a priori themes, while seeking new insights generated from the interviews. Researchers will then code the remaining interviews, comparing themes to distinguish different types of organizations and conditions. To assure coding consistency, every fourth interview will be double-coded. Significant inconsistencies will be discussed and recoded. Finally, we will categorize the themes into a working model.

### Economic analysis

The sustainability of Seva will depend on both its effects and its cost. Our cost analysis is conducted from the adopting FQHC’s perspective and is intended to provide estimates of the costs to implement and operate Seva within an FQHC. The cost data collection instrument follows guidelines for economic evaluation of implementation strategies [54]. It has been adapted from a cost collection instrument we used in a previous quality improvement study [43]. The cost collection instrument will track fixed costs of implementation, including system development costs and research costs related to development of the implementation strategy. The analysis separates these one-time, fixed costs from variable costs related to executing the implementation strategy, including staff salaries (with fringe benefits) for training patients and attending project meetings. We will also track operating costs, such as smartphones, monthly data plans, and IT and clinician time for maintaining and improving Seva. The cost data collection instrument will be administered in semi-annual interviews with staff members from the participating clinics and research team during the intervention period. The coach will also keep a detailed log of contacts with clinic staff to assess staff participation during the intervention. We will estimate the cost of implementing Seva by totaling the time spent by coaches and clinicians during the implementation phase (using coach logs) and then multiplying the hours by relevant wage rates by job classification. We will add any non-personnel costs, such as travel to site visits, the cost of teleconferencing services for follow-up calls, etc. Any free services will be documented. Costs incurred by patients are outside the scope of the evaluation.

Evidence suggests that using Seva in a manner that promotes integrative care will benefit FQHCs financially. Research has shown that drug users who receive regular drug abuse care with regular medical care are less likely to be hospitalized than those who receive only regular medical care alone, regular drug abuse care alone, or neither [55]. For patients with substance-abuse-related medical conditions, integrated care can lower costs through reduced hospitalization rates, inpatient days, and emergency-room use [56]. We will assess changes in the healthcare services provided to patients by collecting healthcare utilization data using a combination of electronic health records and self-report. We will analyze these outcomes using our multiple baseline design to determine whether the costs of implementation are offset by benefits in terms of reduced healthcare utilization.

### Ethics

The study received approval from the Medical Sciences Institutional Review Board at the University of Wisconsin–Madison and is registered at ClinicalTrials.gov (NCT01963234).

### Trial status

At time of submission, the research team is entering the pre-implementation period at the pilot clinic. Implementation of Seva with patients and staff is slated to begin during March 2014.

## Discussion

This research adds to the literature on strategies for integrating addiction treatment into primary care. Our study represents an advanced use of mobile health technology in primary care. Seva may function as a clinician-extender because it can provide much of the education and ongoing therapeutic support that patients with substance use disorders need, allowing clinicians to focus on providing the primary care they are trained to deliver. Seva is unique for giving clinicians a way to regularly monitor their patients’ progress outside the clinic through self-reported substance use, appointment attendance, and risk/protection factors. If implementation proves successful, the field will have a proven strategy for implementing and sustaining an effective technology to help address addiction treatment and recovery support in FQHCs.

### Limitations

Early experience has uncovered a host of unanswered questions related to data sharing, security and interoperability that have been difficult to resolve. For instance, receiving IRB approval for the project was a complex undertaking. Issues related to data sharing are proving to be difficult to standardize from one clinic (and one electronic health record system) to the next. Risk aversion to data sharing may be a formidable challenge to full-scale integration of mobile health technology in primary care. We are currently developing a framework that describes different arrangements for integrating mobile health data into electronic health records, personal health records, mobile devices, and clinical data warehouses.

Our early encounters with FQHCs have revealed organizational differences that will be difficult to account for statistically. For instance, one clinic routinely screens for addiction using standardized screening instruments, while another clinic identifies addiction through conversations with patients. Such pre-implementation differences must be accounted for qualitatively to accurately assess changes in the process of care. If implementation is successful, Seva may help reduce variation in clinics’ approaches to core elements of addiction treatment (including referral and treatment processes), although it is evident that different clinics start from very different places in important respects.

Finally, the patient-level RCTs that form the evidence base for Seva did not include measures of healthcare utilization (*e.g*., ER visits). As we shift towards an organization-level focus in this study of implementation, we seek to understand whether Seva might help patients access health resources more predictably and cost-efficiently. We do not, however, have direct evidence from previous RCTs to support the hypothesis that Seva will reduce healthcare utilization, and analyses related to healthcare utilization should be considered exploratory. Likewise, other outcomes included in our evaluation (for example, quality of life) were unmeasured in the previous RCTs that evaluated TES and A-CHESS. We added these measures after consulting the RE-AIM model dimension checklist (available at http://www.re-aim.hnfe.vt.edu).

## Conclusion

Developing an effective technology for the treatment and continuing care of substance use disorders, and disseminating the technology widely and efficiently, is a matter of urgency given the profound costs of SUDs. Although evidence shows that treatment for substance use disorders has wide-ranging benefits, such care — routine for many other chronic illnesses — is rare in primary care. A model for implementing a mobile technology to support the treatment of substance use disorders could provide a cost-effective means to improve the lives of patients with drug and alcohol problems. Results of the research will be published as they become available.

## Competing interests

Dr. Marsch is affiliated with HealthSim, LLC, a small business that developed the web-based Therapeutic Education System (TES). This relationship is extensively managed by Dr. Marsch and her academic institution. Dr. Quanbeck provides consulting through the NIATx Foundation, a non-profit organization that offers training in quality improvement. All other authors declare that they have no competing interests.

## Authors’ contributions

ARQ drafted the original manuscript. DHG and LAM designed the study. FM, RTB, MLM, JEG, AKA, and HM contributed to the design and conduct of the study. RJ performed critical revisions to the manuscript. All authors read, contributed to, and approved the final manuscript.
